# Comparison of Two Generations of Thoracic Aortic Stent Grafts and Their Impact on Aortic Stiffness in an *Ex Vivo* Porcine Model^☆^

**DOI:** 10.1016/j.ejvsvf.2023.04.001

**Published:** 2023-04-12

**Authors:** Tim J. Mandigers, Michele Conti, Sara Allievi, Francesca Dedola, Daniele Bissacco, Daniele Bianchi, Stefania Marconi, Maurizio Domanin, Joost A. Van Herwaarden, Ferdinando Auricchio, Santi Trimarchi

**Affiliations:** a Department of Vascular Surgery, Fondazione IRCCS Cà Granda Ospedale Maggiore Policlinico, Milan, Italy; b Department of Vascular Surgery, University Medical Centre Utrecht, Utrecht, the Netherlands; c Civil Engineering and Architecture Department, Università Degli Studi di Pavia, Pavia, Italy; d Clinical and Community Sciences Department, Università Degli Studi di Milano, Milan, Italy

**Keywords:** Aortic stiffness, Captivia, Experimental investigation, Navion, Pulse wave velocity, Valiant

## Abstract

**Objective:**

Little is known about the cardiovascular changes after TEVAR and regarding the impact on aortic stiffness for different stent graft generations specifically, following changes in device design. The present study evaluated the stent graft induced aortic stiffening of two generations of the Valiant thoracic aortic stent graft.

**Methods:**

This was an *ex vivo* porcine investigation using an experimental mock circulatory loop. Thoracic aortas of young healthy pigs were harvested and connected to the mock circulatory loop. At a 60 bpm heart rate and stable mean arterial pressure, baseline aortic characteristics were obtained. Pulse wave velocity (PWV) was calculated before and after stent graft deployment. Paired and independent sample *t* tests or their non-parametric alternatives were performed to test for differences where appropriate.

**Results:**

Twenty porcine thoracic aortas were divided into two equal subgroups, in which a Valiant Captivia or a Valiant Navion stent graft was deployed. Both stent grafts were similar in diameter and length. Baseline aortic characteristics did not differ between the subgroups. Mean arterial pressure values did not change after either stent graft, while pulse pressures increased statistically significantly after Captivia (mean 44 ± 10 mmHg to 51 ± 13 mmHg, *p* = .002) but not after Navion. Mean baseline PWV increased after both Captivia (4.4 ± 0.6 m/s to 4.8 ± 0.7 m/s, *p* = .007) and Navion (4.6 ± 0.7 m/s to 4.9 ± 0.7 m/s, *p* = .002). There was no statistically significant difference in the mean percentage increase in PWV for either subgroup (8 ± 4% *vs.* 6 ± 4%, *p* = .25).

**Conclusion:**

These experimental findings showed no statistically significant difference in the percentage increase of aortic PWV after either stent graft generation and confirm that TEVAR increases aortic PWV. As a surrogate for aortic stiffness, this calls for further improvements in future thoracic aortic stent graft designs regarding device compliance.

## Highlights

• Aortic stiffness increases cardiovascular risk and is quantified by aortic PWV.
• Thoracic aortic stent graft deployment has been shown to increase aortic PWV, but intergenerational differences remain unknown.
• This porcine *ex vivo* study did not find a significant difference in the percentage increase of aortic PWV for two generations Valiant thoracic aortic stent grafts.
• Future thoracic aortic stent grafts should aim to improve device compliance, without losing adequate seal and strength.

**What this paper adds**

This experimental study investigated intergenerational differences in thoracic aortic stent graft induced aortic stiffening in an *ex vivo* porcine model. It confirmed that TEVAR increases aortic pulse wave velocity (PWV, m/s) – as a marker of aortic stiffness – and showed that potential improvements in device design do not necessarily result in lower aortic PWV values and higher aortic compliance. This may aid device manufacturers in focusing more on improving future device compliance to prevent potential cardiovascular complications in the long term.

## Introduction

Thoracic endovascular aortic repair (TEVAR) is currently the first choice treatment option for most thoracic aortic diseases according to the most recent clinical practice guidelines of the European and American societies for vascular surgery and is increasingly being adopted to treat more proximal aortic zones.^1^, ^2^, ^3^, ^4^, ^5^

In parallel with these advances, the clinical outcomes of TEVAR are still impaired by several drawbacks of the currently available stent grafts, ranging from device related complications, such as endoleak or migration, to limited long term structural durability.^6^^,^^7^ Moreover, TEVAR has been shown to alter cardiovascular haemodynamics by increasing aortic stiffness^8^ and inducing cardiovascular remodelling over time.^9^^,^^10^ Increased aortic stiffness, normally occurring with age^11^ and quantified by aortic pulse wave velocity (PWV),^12^ is acknowledged to have an important impact on cardiovascular health.^13^

To improve these aspects that may impact the long term outcomes of TEVAR, device manufacturers are constantly developing newer generation stent grafts with improvements in delivery systems, proximal device configurations, or conformability, compared with previous stent graft generations.^14^, ^15^, ^16^, ^17^

Little is known regarding the cardiovascular changes after TEVAR and regarding its impact on aortic stiffness for different stent graft generations specifically, following changes in device design. The aim of the present study was to narrow this gap, by investigating changes in aortic PWV for two generations of the Valiant thoracic aortic stent graft by quantifying their impact on aortic stiffness in an *ex vivo* porcine model. It was hypothesised that a newer generation graft with improved conformability would have less impact on the stent graft induced aortic stiffening.

## Materials and methods

### Aortic specimens

Aortas of young healthy pigs (commercial hybrid, 10–12 months, 160–180 kg) were collected from a local slaughterhouse and evaluated by a veterinary physician to discover potential disorders. The aortas were procured from the aortic valve to the renal arteries. No pigs were sacrificed solely for the purpose of this study but were raised for commercial purposes. Therefore, ethical approval by the local animal ethics committee was waived. Preservation and transportation took place in 0.9% saline solution at 4°C and the experiments were conducted within 48 hours of harvesting to ensure the freshness of the specimens. Before the experiment, each aortic specimen was surgically prepared from the aortic root to the coeliac trunk at room temperature, by removing excess connective and cardiac tissue. Side branches (e.g., spinal arteries and the two supra-aortic trunks) were ligated. Any small iatrogenic transmural lesion caused during preparation, this was sutured with Prolene 4–0.

### Experimental set up

**Figure 1:**
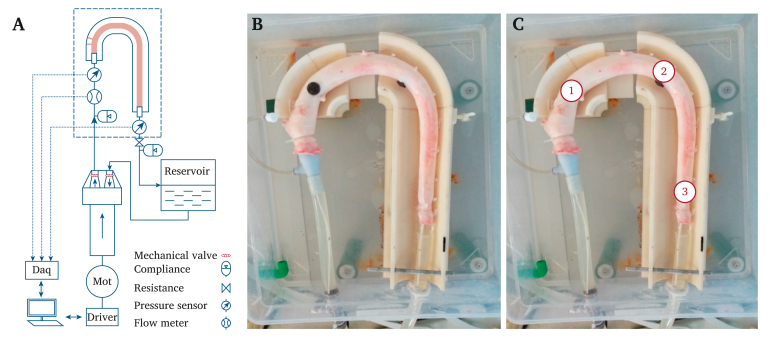
(A) Schematic representation of the mock circulatory loop and its components. Daq = data acquisition; Mot = motor (B) Porcine aorta connected to the mock circulatory loop (C) Schematic representation of the three pre-defined points where the aortic diameters were measured. 1. just distal to the second porcine supra-aortic trunk; 2.10 cm distal to point 1; 3. just before the distal tube connector. The proximal stent graft edge was deployed just distal to the second porcine supra-aortic trunk, from point 1 to point 2. Table 1 provides the corresponding aortic diameters.

The aortas were connected to a circulatory mock loop, which allowed for intraluminal pressurisation under continuous steady state or pulsatile flow in a controlled manner (Figure. 1A). Steady state flow was obtained with a centrifugal pump (Medtronic Biomedicus 550, Minneapolis, MN, USA), while pulsatile flow was obtained with a custom made pulsatile pump containing both mechanical heart valves.^18^ The pulsatile pump was set at a heart rate of 60 beats per minute and cardiac output of 4.5 litres per minute. Peripheral resistance was set to obtain a mean arterial pressure (MAP) between 80 and 100 mmHg within the aortic specimens of every experiment. A 3D printed case guided the aortic specimens to approximate the movement of the thoracic aorta within the thoracic cavity, as shown in Figure 1B. Water was kept at body temperature with a liquid heater (Schego 542 Heizer Titan [100 Watt], Offenbach am Main, Germany) and was used for perfusion to preserve the biomechanical characteristics of nitinol stents and to prevent tissue dehydration. Intraluminal pressures were recorded constantly with two pressure sensors (Honeywell pressure sensor 40pc015g series, Morristown, NJ, USA) located in the ascending aorta and just above the coeliac trunk 1 cm from the connection of the aorta to the silicone tubes. Pressures were recorded for at least 10 consecutive cardiac cycles, after stable values were obtained.

### Aortic measurements

Prior to pressure measurement under pulsatile flow, the specimens were pressurised up to a MAP of 80–100 mmHg by steady state flow, to repair secondary leakage and to measure luminal calibres. Pulse pressure (PP) was measured, defined as the difference between systolic and diastolic blood pressures. Baseline diameters were measured manually with an ultrasound probe (Medison Accuvix XQ, Seoul, South Korea) and by two skilled operators (S.A., D.B.). Measurements were performed from adventitia to adventitia. The plastic box in which the 3D printed case, the porcine aorta, and the silicone connecting tubes were positioned, was filled with water to act as echocontrast media. Diameters were collected at three predefined points, the first at the proximal landing zone just distal to the second porcine supra-aortic trunk, the second 10 cm distal to point 1, and the third just before the distal tube connector (Figure. 1C). Aortic centreline length measurements were performed using open source image processing and measurement software (ImageJ, U.S. National Institutes of Health, Bethesda, MD, USA). A planar image of the aortic specimen, taken with a digital camera parallel to the aortic plane, was imported to the ImageJ software. Pixels were scaled in millimetres using a reference of2 cm in the image (Figure. 1B). Following calibre measurements, steady state flow was replaced by pulsatile flow and aortic PWV measurements were performed, as a surrogate for aortic stiffness.

### Stent graft devices and implantation

Two different stent graft types were deployed, the earlier generation (second) Captivia (Medtronic, Minneapolis, MN, USA) and the newer generation (third) Navion (Medtronic, Minneapolis, MN, USA). Stent graft size for Captivia used in the present study was 26–26-100, and 25-25-96 for Navion. This study started before the global device recall for Navion, and the decision to continue the analysis was taken to better understand the potential improvements of newer generation stent grafts in terms of device compliance.

After distal disconnection of the aorta from the circulatory mock loop, stent grafts were deployed using a custom made delivery system (Appendix A). The proximal stent graft edge was deployed just distal to the second porcine supra-aortic trunk from point 1 to point 2 (Figure 1C). After deployment and reconnection of the aorta to the loop, the proximal and distal landing zones were confirmed manually. Intraluminal pressures were recorded at the level of the ascending aorta and just above the coeliac trunk. Aortic PWV (in metres per second [m/s]) was calculated by dividing the distance between the tips of the proximal (ascending aorta) and distal (just above the coeliac trunk) pressure sensors, by the time between the two minima of the proximal and distal pressure signals (transit time [TT]), following the foot to foot method. The same stent graft was reloaded into the custom made delivery system and used for the next experiment (Appendix A).

### Data analysis

Boxplots were created to summarise results graphically. Exclusion criteria were adopted: (1) a conservation time of more than 48 hours between harvesting of the aorta and the experiment; (2) aortic specimens with severe aortic leakage during continuous flow pressurisation; (3) initial technical issues that resulted in unstable pressure values during continuous and/or pulsatile pressurisation; (4) experiments with a decline in PWV after stent graft deployment were not considered for statistical analysis as the impact of a stent graft on aortic PWV can be zero at minimum from a theoretical biomechanical point of view;^8^^,^^9^^,^^19^, ^20^, ^21^, ^22^ (5) extreme PWV increase outliers (>Q3 + 3 ∗ interquartile range) after stent graft deployment. Data were analysed using Matlab version R2022b (Mathworks, Natick, MA, USA), Microsoft Excel (Microsoft, Redmond, WA, USA), and IBM SPSS Statistics versions 27 and 28 (SPSS Inc., Chicago, IL, USA). Data are reported as number (*n*) and percentage (%), or as mean ± standard deviation (SD). The Shapiro–Wilk test was performed to test for normality. Independent samples *t* test and paired student *t* test were performed to compare independent and paired groups of normally distributed measurements, respectively. In the case of non-normally distributed data, non-parametric alternatives Wilcoxon rank sum and Wilcoxon signed rank tests were performed. Two sided *p* values < .050 were considered statistically significant. Intra-observer, interobserver agreement, and repeatability coefficients (RC, reported as number and percentage of the mean of all measurements) were assessed for the centreline length (TM, DB) and (manually adjusted) transit time (TT) measurements (MC, DB), according to the Bland-Altman method (see Appendix B for a detailed explanation).^23^

## Results

In total, 31 porcine aortas were harvested and connected to the pulsatile mock circulatory loop between July 2020 and November 2021. Captivia was deployed in 16 aortas (52%), and Navion in 14 (45%). One aorta (3%) was excluded before stent graft deployment due to excessive leakage during pressurisation. Four initial samples (13%) were excluded due to technical issues, and one (3%) due to a conservation time > 48 hours. Exclusion criteria 4 and 5 led to an inclusion range of PWV changes after stent graft deployment from 0% to 21.8% (for the Captivia subgroup). In the remaining 25 experiments (81%), this led to four (13%) exclusions due to a decline in PWV after stent graft deployment (Captivia subgroup: *n* = 3, Navion subgroup: *n* = 1), while one (3%) was an extreme PWV increase outlier (Captivia subgroup).

Consequently, 20 experiments were found to be eligible for the present analysis, and the Captivia subgroup (*n* = 10) was compared with the Navion subgroup (*n* = 10). Baseline aortic specimen characteristics are shown in Table 1. The porcine thoracic aortas were tapered from proximal to distal (Table 1). Therefore, oversizing at the proximal landing zone (PLZ) in the Captivia subgroup was 6% ± 8%, gradually increasing to a distal landing zone (DLZ) oversizing of 34% ± 9%. Similarly, in the Navion subgroup, PLZ oversizing was 6% ± 7%, gradually increasing to a DLZ oversizing of 32% ± 11%. There was no statistically significant difference regarding the oversizing at the PLZ and DLZ between stent grafts (PLZ: *p* = .96, DLZ: *p* = .66). In 17 (85%) specimens, experiments were conducted within 24 hours of harvesting and in the remaining three (15%) within 48 hours.

**Table 1:** Baseline aortic specimen characteristics and differences between the Captivia and Navion subgroups.

	Captivia subgroup (*n* = 10)	Navion subgroup (*n* = 10)	*p*
Diameter point 1∗ – cm	2.5 ± 0.2	2.4 ± 0.2	.24
Diameter point 2∗ – cm	2.0 ± 0.1	1.9 ± 0.2	.54
Diameter point 3∗ – cm	1.7 ± 0.1	1.7 ± 0.2	.29
Centreline length – cm	31.2 ± 3.3	33.0 ± 3.6	.25
Conservation time – d	1.20 ± 0.42	1.10 ± 0.32	.74

Data are presented as mean ± SD.

∗ See Figure 1C for a schematic specification of the locations of porcine aortic diameter measurements.

**Figure 2:**
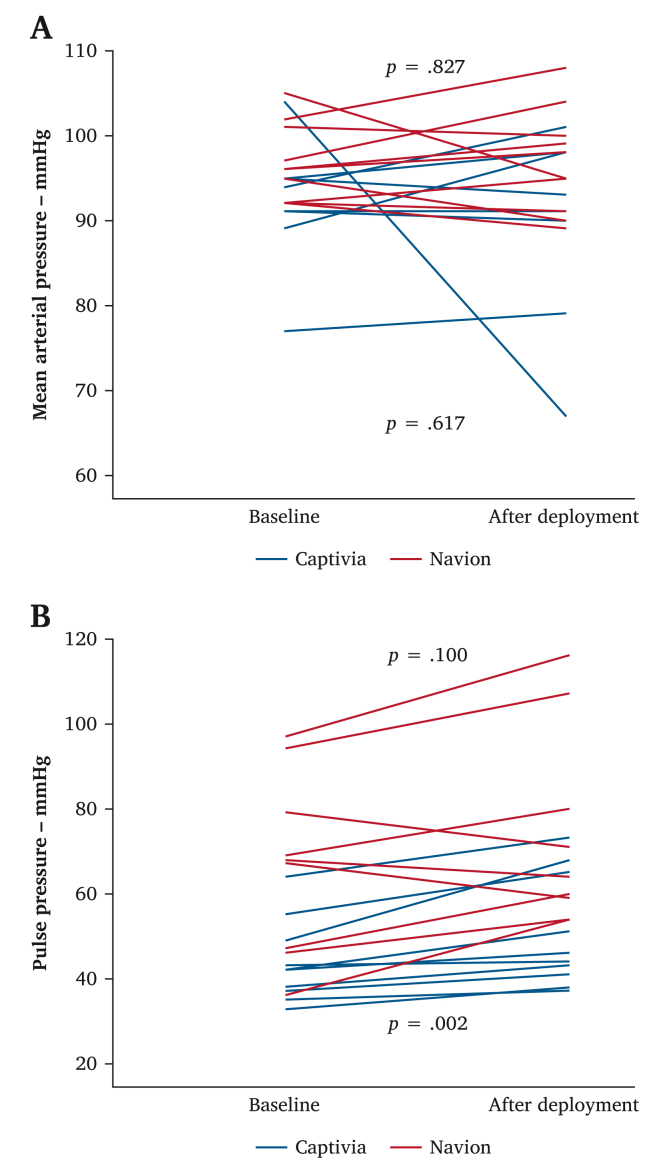
Spaghetti plots of the changes in mean arterial pressure (A) and pulse pressure (B) before and after stent graft deployment.

In both subgroups, MAP values did not significantly change after Captivia (mean MAP from 92 ± 7 mmHg to 90 ± 10 mmHg, *p* = .62) and Navion (mean MAP from 97 ± 4 mmHg to 97 ± 6 mmHg, *p* = .87) deployment. A statistically significant increase was found in PP after Captivia (mean PP from 44 ± 10 mmHg to 51 ± 13 mmHg, *p* = .002) but not after Navion (mean PP from 68 ± 20 mmHg to 74 ± 22 mmHg, *p* = .100) deployment. Figure 2 shows the MAP and PP changes for both subgroups.

**Figure 3:**
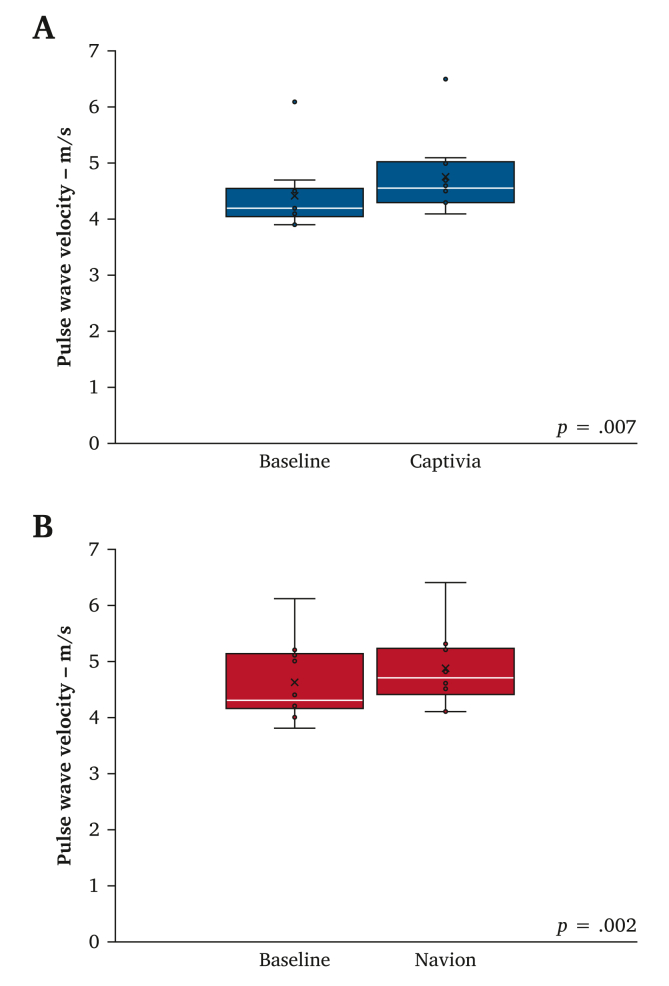
(A) Boxplot of the aortic pulse wave velocity values before and after Captivia deployment (*n* = 10) (B) Boxplot of the aortic pulse wave velocity values before and after Navion deployment (*n* = 10).

**Figure 4:**
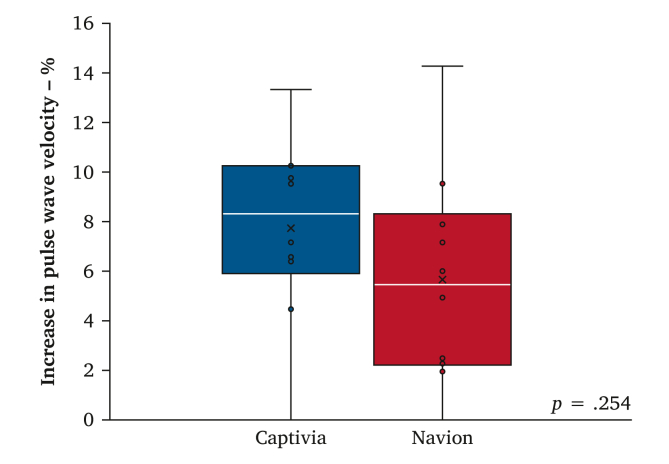
Boxplot of the percentage increase in aortic pulse wave velocity for the Captivia (*n* = 10) and Navion (*n* = 10) subgroups.

Baseline aortic PWV did not differ between the Captivia and Navion subgroups (Table 2). Boxplots of the PWV values before and after stent graft deployment are shown in Figure 3, and a substantial increase was found in PWV in both subgroups. A boxplot of the % increase in PWV for both subgroups is shown in Figure 4. A lower mean % increase was found in PWV after Navion compared with Captivia; however, this finding was not statistically significant (Table 2).

**Table 2:** Differences between the Captivia and Navion subgroups regarding baseline pulse wave velocity and the pulse wave velocity (PWV) after stent graft deployment.

	Captivia subgroup (*n* = 10)	Navion subgroup (*n* = 10)	*p*
Baseline PWV – m/s	4.4 ± 0.6	4.6 ± 0.7	.48
PWV after stent graft deployment – m/s	4.8 ± 0.7	4.9 ± 0.7	–
% increase in PWV after stent graft deployment – m/s	8 ± 4	6 ± 4	.25

Data are presented as mean ± SD.

Intra-observer and interobserver agreement for the centreline length measurements (*n* = 20) and TT measurements (*n* = 5) was adequate (Appendix B). For the centreline length measurements, the intra-observer RC was .86 cm (3%) and interobserver RC was .68 cm (2%).

## Discussion

This *ex vivo* study evaluated changes in aortic PWV, a marker of aortic stiffness, after deployment of two generations of Valiant thoracic aortic stent grafts in thoracic porcine aortas connected to a mock circulatory loop. To the present authors’ knowledge, this is the first study to have investigated differences in stent graft induced aortic stiffening between two generations of thoracic aortic stent grafts in an experimental setting, following improvements in device conformability. The main finding is no statistically significant difference in the percentage increase of aortic PWV after deployment of the stent grafts (Figure. 4). Moreover, it is confirmed that aortic PWV increases after TEVAR with both devices (Figure. 3).

Potential improvements in device design may reduce the impact of thoracic aortic stent grafts on aortic stiffness and prevent future cardiovascular events.^8^^,^^9^^,^^13^^,^^24^ This may improve the long term outcomes of endovascular aortic treatment modalities by reducing a patient's cardiovascular risk. On the other hand, caution and lifelong surveillance remain crucial, as this may also negatively impact clinical outcomes or cause device failure.^7^ Reasons for the global device recall of Navion were 11 structural failures between one and four years of follow up (e.g., type IIIb endoleaks, fractures and loss of seam integrity, stent ring enlargement).^7^

The present findings are comparable with previous porcine *ex vivo* studies and show a similar order of magnitude in mean PWV increase (range 4–9%).^21^^,^^22^ One of these studies only found a statistically significant increase in PWV after distal extension of a single stent graft (length 100 mm), suggesting that the increase in PWV might be dependent on the amount of aortic coverage by TEVAR.^21^ In contrast to this, the main findings of the present study and of another study that compared four different stent graft brands showed an increase in aortic PWV after deployment of a single stent graft with 96–100 mm aortic coverage.^22^ However, in the same study it was concluded that the increase in aortic PWV was dependent on the extent of stent graft coverage.^22^

After Captivia deployment, a statistically significant increase was found in PP, while this was not found after Navion deployment (Figure. 2B). This may draw attention to the fact that different devices could impact cardiovascular haemodynamics in different ways. In humans, certain physiological compensation mechanisms may mitigate these effects. Increases in systolic blood pressure or PP following increases in aortic stiffness causes increased pulsatile damage to target organs, especially those that operate at high arterial flow and low vascular resistance (e.g., kidneys, brain).^13^^,^^25^^,^^26^ Here, it seems important to note that natural aortic stiffening occurring with ageing and an acutely induced aortic stiffness mismatch after stent graft deployment are two different things. Nevertheless, they both increase aortic PWV, and the haemodynamic impact seems comparable from a conceptual point of view. Moreover, due to increased aortic stiffness, cardiac afterload increases, and coronary perfusion pressure reduces. This has been shown to induce adverse cardiac and aortic remodelling over time by several clinical, experimental, and computational investigations.^9^^,^^10^^,^^19^^,^^20^^,^^27^

Altogether, there is a growing interest in evaluating the long term outcomes of TEVAR for different aortic diseases. Adverse outcomes may be of specific importance in young patients without comorbidities, typically treated with TEVAR for blunt thoracic aortic injury (without questioning the application of TEVAR to treat this life threatening disease).^9^ Research regarding this topic need to be advocated as it can provide useful insights for physicians to improve the clinical outcomes of TEVAR, and it could aid medical device manufacturers with future stent graft development. Moreover, as the general treatment trend is shifting towards the endovascular management of arterial and venous disease, related issues such as aortic stiffening or more widely vascular stiffening, request knowledge, attention, and a specific approach.

### Limitations

*Ex vivo* studies investigating the biomechanical coupling between TEVAR and porcine aortic tissue have inherent limitations. The mock circulatory loop aims at eliminating factors that could influence the results, such as variations in blood pressure, as PWV is known to be dependent on MAP.^12^^,^^28^ Future development of the set up would aim to integrate the control of both baseline MAP and PP. On the one hand, this experimental setting allows for control, isolation, and analysis of certain parameters, while there is variability in other parameters at the same time (e.g., aortic specimens). This is the main reason for the relatively high number of exclusions in which a PWV decline after stent graft deployment (*n* = 4) or extreme PWV increase (*n* = 1) was found, compared with the other experiments. Moreover, sample size calculation was not performed for the primary outcome, which might have led to a false acceptance of the null hypothesis (type II error). Next, thoracic porcine aortic tissue is most comparable with human aortic tissue < 60 years old, and the results of the present study might thus be less translatable to patients > 60 years old.^29^ A single stent graft size was used in both subgroups, and this stent graft was not gradually tapered to have an equal amount of oversizing at the PLZ and DLZ. The slight difference in diameter (1 mm) and length (4 mm) between both stent grafts may theoretically have introduced a bias on the results. As mentioned by previous authors, water is known to have a lower viscosity than blood but is a commonly used perfusion fluid in *ex vivo* porcine models.^30^ The influence on PWV measurements is expected to be low due to the high speed of travel of water in a pulsatile environment.^31^ Another possible limitation might be that the porcine aortas had no surrounding connective tissues as in humans, and this might influence movement or passive biomechanics.^32^

### Future directions

Potential tools like 3D printing and *in silico* simulations may add value to these experiments.^33^ They could aid the development of materials that most closely mimic the mechanical properties of the native aorta, reducing their impact on aortic stiffness and thus cardiovascular health. The need to develop a more compliant stent graft, without losing adequate sealing and strength, is further underlined by the main findings of this study. The use of porcine aortas might be reduced if the use of *in silico* models can be validated against porcine or cadaveric aortic tissue.^34^^,^^35^ Moreover, the experimental model allows investigation of the impact of different aortic arch geometries or open surgical aortic repairs on aortic PWV.

### Conclusions

This porcine *ex vivo* study did not find a statistically significant difference in the percentage increase of aortic PWV of two generations of Valiant thoracic aortic stent grafts; however, both stent grafts increased aortic PWV, as a surrogate for aortic stiffness.

## Funding

Thoracic aortic stent grafts used in the present study were provided by Medtronic, Inc.

## Conflict of interest

Joost A. Van Herwaarden is or has been proctor or consultant for Medtronic, Gore Medical, Terumo Aortic and Cook Medical. Santi Trimarchi is consultant and speaker for Medtronic Inc., WL Gore., and Terumo Aortic.
