# Supplementary material for: Comparison of Two Generations of Thoracic Aortic Stent Grafts and Their Impact on Aortic Stiffness in an Ex Vivo Porcine Model
Source: EJVES Vasc Forum. 2023 Apr 12;59:8–14. doi: 10.1016/j.ejvsvf.2023.04.001 (PMC10199196; doi:10.1016/j.ejvsvf.2023.04.001)
Supplement: Multimedia component 1 [file mmc1.pdf]

Appendix A.

Components of the delivery system

As illustrated in Figure 1, the delivery system is composed by the following parts: (1,2) main body acting as a case embracing the handling system (3,4) and the guide (5) of the inner bar (6) with a distal metallic crown to hold the endograft's proximal Z-shaped nitinol ring tips; (7) the outer shaft, where the endograft is inserted; (8) a funnel to support the retrieval of the endograft within the outer shaft during the loading stage. All the components besides the metallic bar of part (6) are 3D printed using HP MultiJet Fusion 580 Color.

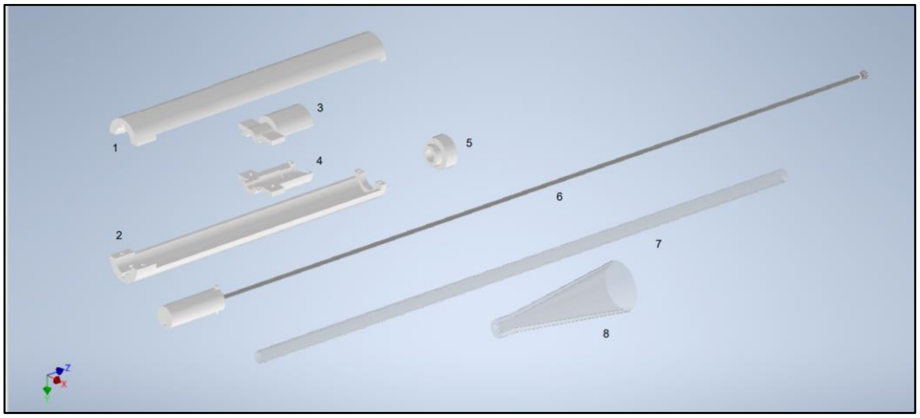

Figure 1. CAD drawing of the delivery system components: main body (1,2); handling system (3,4); guide (5) of the inner bar (6); outer shaft (7); loading funnel (8).

Endograft loading into delivery system and deployment.

As illustrated in Figure 2, by moving the handle forward, to which the outer shaft and the funnel are connected, the endograft is compressed and loaded. The size of the stent will reduce as it passes through the funnel (image B highlights this process). Once inside the flexible tube, the funnel can be removed, and the tips of the free-flow rings links are fixed to the metallic crown located in the distal part of the inner bar.

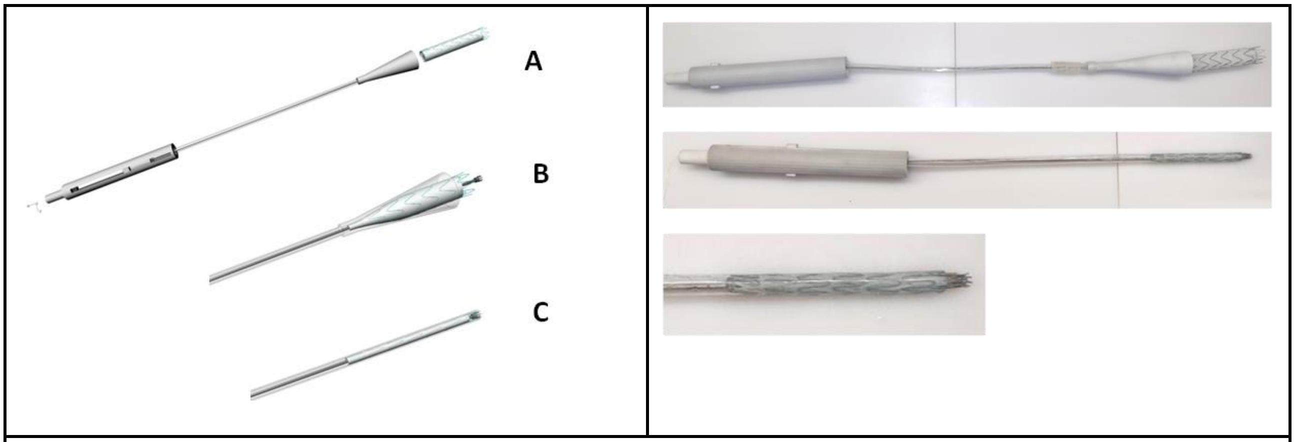

Figure 2. On the left. Sequence of endograft loading within the delivery system. (A) Initial alignment of the system and the endograft; (B) partial insertion of the endograft into the outer shaft through the compression facilitated by the conical funnel; (C) endograft fully inserted into the delivery system.

On the right, picture of the actual delivery system and the endograft, with a zoom on the loaded endograft inside the outer shaft.

The endograft is then released by moving the handle backwards. By doing this, the tube will follow the path of the handle and then gradually release the endograft.

Appendix B.

Aspects of the manuscript susceptible to variability

Two aspects included in the calculation of our primary outcome (i.e., aortic pulse wave velocity [PWV, m/s]), are susceptible to variability because they are operator dependent, namely the centreline length measurements and transit-time (TT) measurements (partially). As specified in the Materials and Methods section, aortic PWV = 1 / 2. Please see below:

1. Aortic specimen centreline length (cm): corresponding to the distance between the proximal and distal pressure sensor in the ascending aorta and just above the celiac trunk. Measured using an open-source image processing and measurement software (ImageJ, U.S. National Institutes of Health, Bethesda, MD, U.S.A.).
2. Transit-time (seconds): the time between the two minima (feet) of the proximal and distal pressure signals. To achieve this value, the detection of the pressure wave foot is crucial. We have processed the acquired inlet and outlet pressure curves, using Matlab R2020b (Mathworks, Natick, MA, U.S.A.), as follows:
    - The two pressure curves are synchronized in time, using the trigger signal of the pump engine of the pulse duplicator;
    - The time synchronization allows to split the signals according to the beats;
    - Ten beats are selected along the entire acquisition and an average pressure curve is generated by averaging the data of the selected beats;
    - The average curve is smoothed by a moving average filter with default settings;
    - Time derivate of the pressure curve is computed;
    - The initial (mechanical) guess of the pressure curve foot is done using the value of maximum derivative, i.e., the time point where the rate of pressure change is highest (see Figure 1).
    - To avoid errors due to pressure curve oscillation, the automatic guess is then visually inspected by the user who can confirm the suggestion or refuse it and then manually pick the foot point by a graphical user interface (see Figure 2).

Figure 1. The initial mechanical foot selection (blue dot) is accepted by the user.

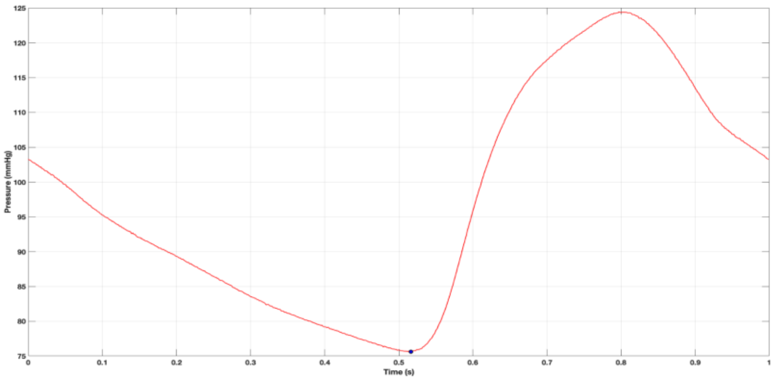

Figure 2. The initial mechanical guess (red dot) is refused, and the user selects the minimum of the pressure curve (blue dot).

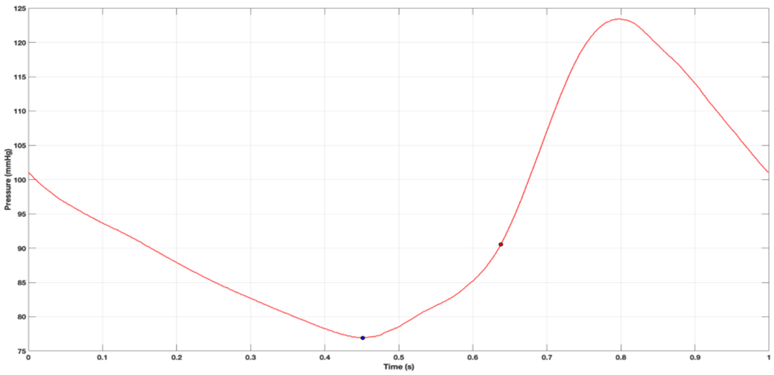

Intraobserver and interobserver variability

Intra and interobserver variability was assessed for all aortic specimens (n = 20) and for a subset of the pressure curves (n = 5) since most of these were selected mechanically (as specified above).

Measurements were performed under the same conditions, using the same method, and with a one-week interval. Different measurements were compared using a paired student's t-test. Intraobserver, interobserver agreement and repeatability coefficients were assessed following the Bland-Altman method[1]:

- Bland-Altman plots were created, plotting the differences between paired measurements against the mean of paired measurements. The centre of agreement was set at the mean of the differences between paired measurements. The upper and lower limits of agreement were set at 1.96*SD above and below this mean.
- Repeatability coefficients (RC) were calculated and presented as number and percentage of the mean of the measurements.

Intraobserver and interobserver variability of length measurements

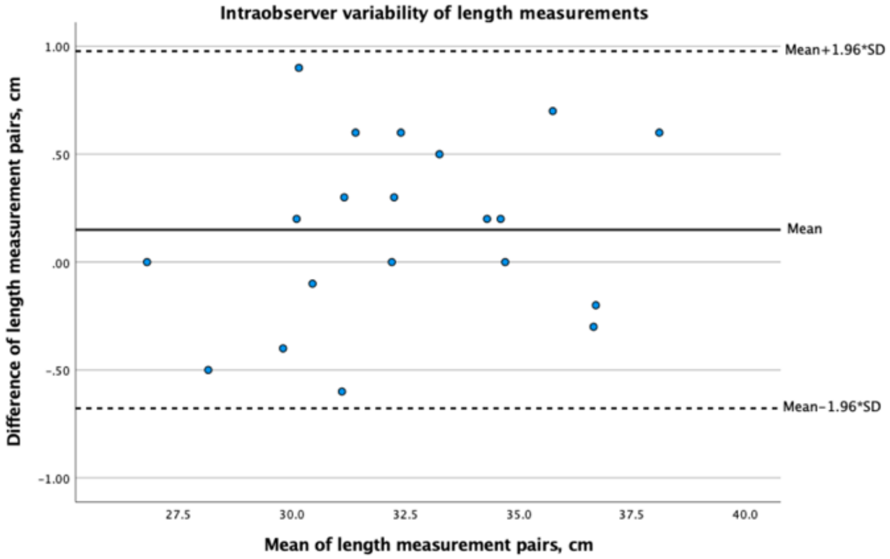

There was no significant difference between the measurements of the observer (p = .129). There were no measurements outside the two limits of agreement. RC: .86 cm (3%).

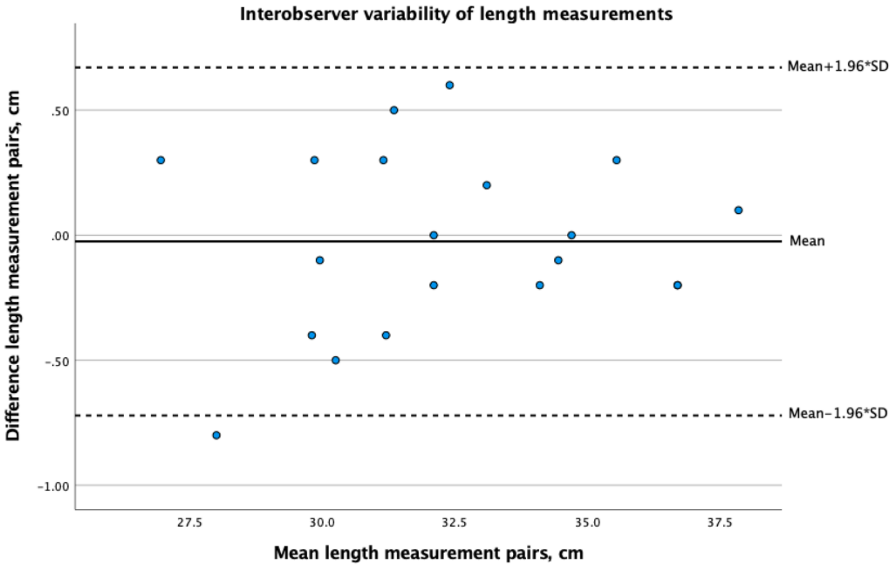

There was no significant difference between the measurements of the observer (p = .756). There was one measurement below the lower limit of agreement. RC: .68 cm (2%). Linear regression through the Bland-Altman scatter plots was not significant (intraobserver: p = .357; interobserver: p = .601), suggesting the absence of proportional bias.

Intraobserver and interobserver variability of transit-time (TT) measurements

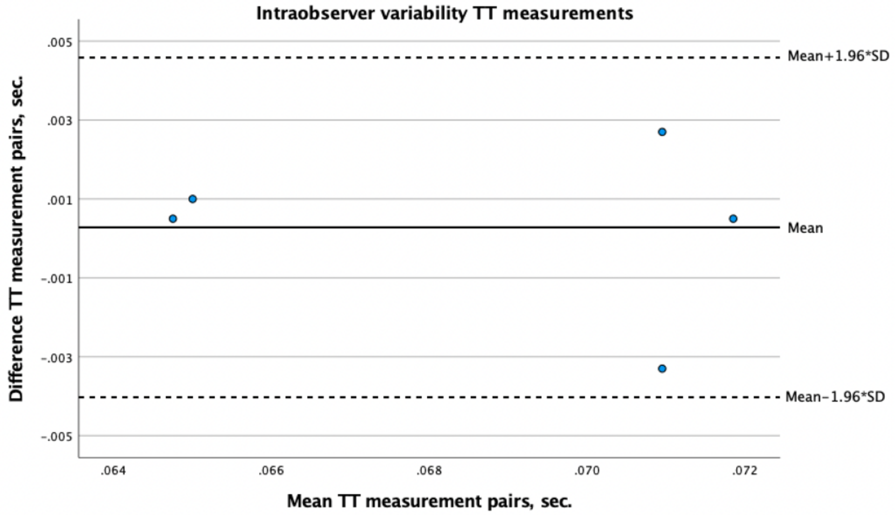

There was no significant difference between the measurements of the observer (p = .790). There were no measurements outside the two limits of agreement. RC: .002 seconds (3%).

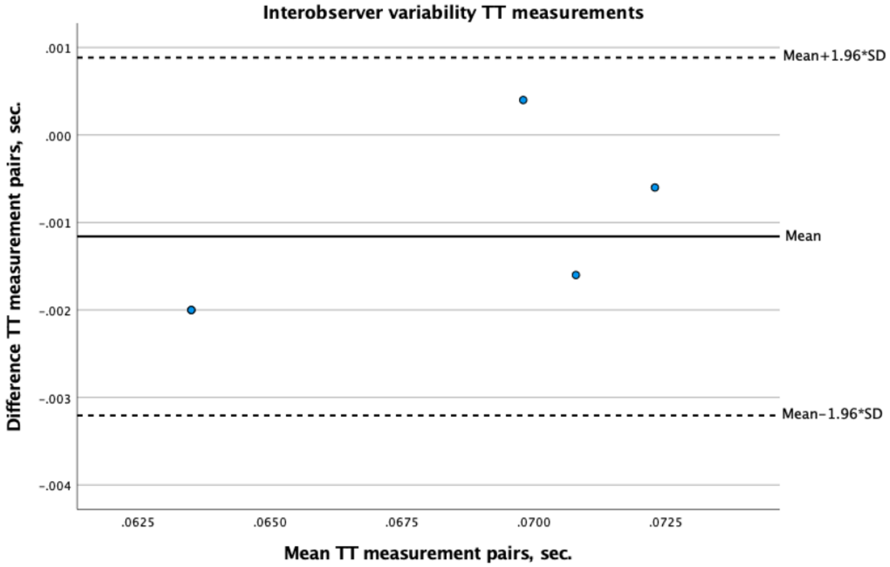

There was no significant difference between the measurements of the observer (p = .068). There were no measurements outside the two limits of agreement. RC: .003 seconds (4%). Linear regression through the Bland-Altman scatter plots was not significant (intraobserver: p = .776; interobserver: p = .224), suggesting the absence of proportional bias; however, there is a limited number of measurements (n = 5).

References

1. Bland JM, Altman DG. STATISTICAL METHODS FOR ASSESSING AGREEMENT BETWEEN TWO METHODS OF CLINICAL MEASUREMENT. Lancet. 1986 Feb 8;1(8476):307-10. PMID: 2868172.
